# Robotic-assisted anatomic anterior cruciate ligament reconstruction: a comparative analysis of modified transtibial and anteromedial portal techniques in cadaveric knees

**DOI:** 10.3389/fbioe.2024.1360560

**Published:** 2024-03-06

**Authors:** Ling Zhang, Jinpeng Lin, Xuan Zhao, Mingwei Liu, Yao Hou, Yu Zhang, Jinzhong Zhao, Shaobai Wang

**Affiliations:** ^1^ School of Exercise and Health, Shanghai University of Sport, Shanghai, China; ^2^ Department of Orthopaedics, Guangdong Provincial Hospital, Guangdong Academy of Medical Sciences, Guangzhou, China; ^3^ Shanghai Droidsurg Medical Technology Co., Ltd., Shanghai, China; ^4^ Department of Sports Medicine, Shanghai Sixth People’s Hospital, Shanghai Jiao Tong University, Shanghai, China

**Keywords:** anatomic reconstruction, transtibial, anteromedial portal, tunnel length, obliquity

## Abstract

**Introduction:** This study employed surgical robot to perform anatomic single-bundle reconstruction using the modified transtibial (TT) technique and anteromedial (AM) portal technique. The purpose was to directly compare tunnel and graft characteristics of the two techniques.

**Methods:** Eight cadaveric knees without ligament injury were used in the study. The modified TT and AM portal technique were both conducted under surgical robotic system. Postoperative data acquisition of the tunnel and graft characteristics included tibial tunnel position, tunnel angle, tunnel length and femoral tunnel-graft angle.

**Results:** The mean tibial tunnel length of the modified TT technique was significantly shorter than in the AM portal technique (*p* < 0.001). The mean length of the femoral tunnel was significantly longer for the modified TT technique than for the AM portal technique (*p* < 0.001). The mean coronal angle of the tibial tunnel was significantly lower for the modified TT technique than for the AM portal technique (*p* < 0.001). The mean coronal angle of the femoral tunnel was significantly lower for the AM portal technique than for the modified TT technique (*p* < 0.001). The AM portal technique resulted in a graft bending angle that was significantly more angulated in the coronal (*p* < 0.001) and the sagittal planes (*p* < 0.001) compared with the modified TT technique.

**Discussion:** Comparison of the preoperative planning and postoperative femoral tunnel positions showed that the mean difference of the tunnel position was 1.8 ± 0.4 mm. It suggested that the surgical navigation robot could make predictable tunnel position with high accuracy. The findings may support that the modified TT technique has benefits on femoral tunnel length and obliquity compared with AM portal technique. The modified TT technique showed a larger femoral tunnel angle in the coronal plane than the AM portal technique. Compared with the modified TT technique, the more horizontal trajectory of the femoral tunnel in the AM portal technique creates a shorter femoral tunnel length and a more acute graft bending angle.

## 1 Introduction

The arthroscopic reconstruction is a common treatment for anterior cruciate ligament (ACL) injury (Chin et al., 2019). The goals of ACL reconstruction are to restore knee stability and to regain full knee function. The success of ACL reconstruction depends on a variety of factors, and tunnel placement plays one of the most significant roles in restoring knee stability (Strauss et al., 2011; Uliana et al., 2012). Tunnel malposition is the most common technical error leading to graft failure (Christensen et al., 2018). The revision rate after ACL reconstruction ranges from 10% to 40%, of which 70%–80% are because of tunnel misplacement (Chin et al., 2019). Over the past decades, anatomic ACL reconstruction has been advocated to ensure ideal function of the reconstructed ACL, resulting in better biomechanical and clinical outcomes. To improve patient outcomes, surgeons should consider factors such as graft choice, proper surgical technique, and patient-specific risk factors when performing ACL repairs.

Transtibial (TT) technique is one of the most common approaches for single-bundle (SB) ACL reconstruction (Brophy and Pearle, 2009; Zaffagnini et al., 2011). In the TT technique, the femoral drill guide is inserted through the tibial tunnel, so the femoral tunnel position is dependent on the orientation of the tibial tunnel. However, numerous studies have shown that the traditional TT technique failed to place the anatomic femoral tunnel (Zbek and Binnet, 2021). Hence, independent drilling of the femoral tunnel using the anteromedial (AM) portal technique has become an alternative to the TT technique for anatomic SB ACL reconstruction (Chang et al., 2011; Youm, 2014). For the AM portal technique, the femoral drill guide is inserted through the medial portal, which enables the surgeon to position the femoral tunnel independently of the tibial tunnel. However, this may be accompanied by the greater risks of posterior wall blowout, short femoral tunnels and acute graft bending angle (Chang et al., 2011).

As a result of these limitations, new surgical techniques or practices have been developed to improve patient outcomes and reduce graft failure in ACL repairs. Some investigators modified the TT technique to achieve anatomic ACL reconstruction but this is much more technically challenging (Piasecki et al., 2011; Trofa et al., 2020). Despite various studies demonstrating the appropriate tibial tunnel starting position and angle for anatomical TT reconstruction (Trofa et al., 2020), it is still difficult to achieve an anatomic tunnel position using handheld locators under arthroscopy. The surgeon determines the tunnel position only through subjective observation during arthroscopic reconstruction, and the positioning accuracy is affected by the surgeon’s learning curve (Park et al., 2016). However, intraoperative placement errors of the tunnel can be reduced with the assistance of surgical navigation robots (Endele et al., 2009; Zhu et al., 2013). Robotic surgery has been found to reduce blood loss, transfusion rates, length of hospital stay, and overall complication rates compared to traditional surgical methods (Davies et al., 2015). Five-year survival rates after robotic surgery exceeding 95% (Davies et al., 2015). A study comparing patient satisfaction between robotic-assisted surgery and traditional surgery found higher satisfaction with robotic surgery (Xue et al., 2024). Furthermore, orthopaedic surgical navigation robots provide the possibility of anatomical reconstruction with a modified TT (Ding et al., 2022).

Therefore, this study employed surgical navigation robot to perform anatomic SB ACL reconstruction using the modified TT technique and AM portal technique. The purpose was to validate the accuracy of the femoral tunnel created by surgical navigation robot, and to directly compare tunnel and graft characteristics of the two techniques, including the tunnel position, tunnel angle, tunnel length and graft obliquity. We hypothesized that the modified TT technique performed by robotic system would achieve anatomic femoral tunnel with longer femoral tunnel length and less tunnel-graft bending angle compared with the AM portal technique.

## 2 Materials and methods

Eight cadaveric knees without ligament injury were used in the study. This study used strains obtained from human cadaver samples. Institutional ethics committee of Shanghai Sixth People’s Hospital did not require the study to be reviewed or approved by an ethics committee because they were deidentified. All included knees had no evidence of degenerative arthritis, ligament injuries or prior surgeries. The skin and muscles were sharply resected, leaving the cruciate ligaments, capsule, collateral ligaments, and menisci intact. The tibia, fibula, and femur were transected, leaving a length of 25 cm for each bone. Before testing, each cadaveric knee was thawed at room temperature for 24 h. During the testing, each specimen was kept moist with 1.0% saline solution. The medial parapatellar arthrotomy was performed and the intact ACL was sectioned at femoral and tibial insertions, leaving 1–2 mm soft tissue footprint. These were done in each cadaveric knee.

### 2.1 Intraoperative acquisition of specific landmarks

**FIGURE 1:**
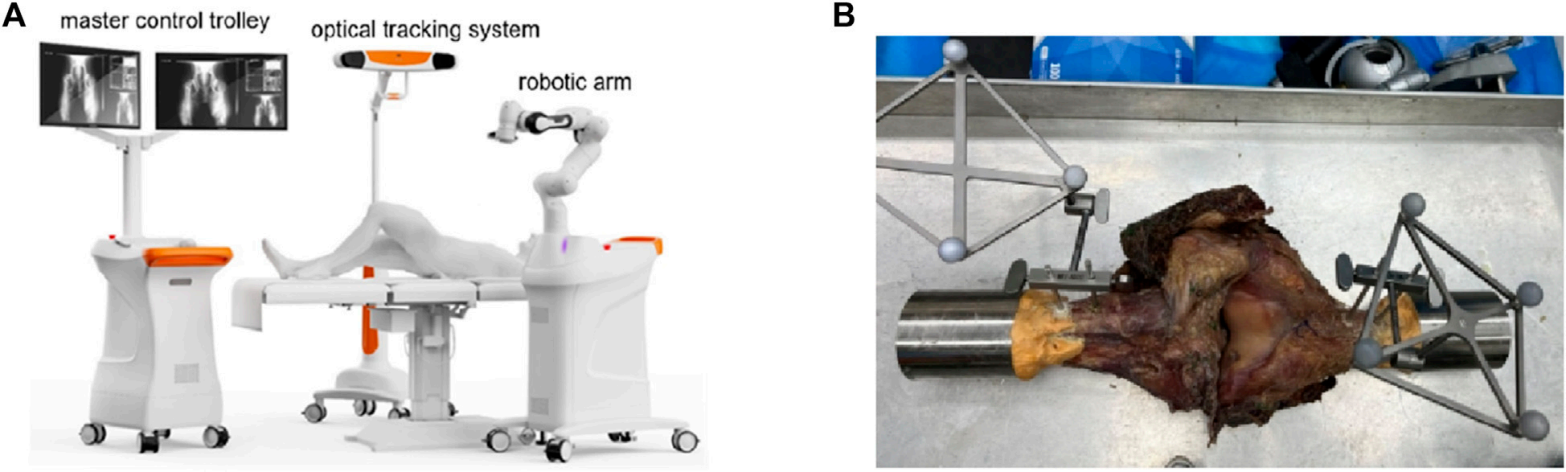
**(A)** The overall composition of Intelligent Knee Stability Restoration; **(B)** Two rigid bodies fixed to the tibia and femur at full extension.

The knee was placed on the operating table at full extension. The constraints of the collateral ligament, posterior cruciate ligament, and congruity of the articular surfaces all contributed to a neutral knee position. Navigation was performed based on an image-free technique usingIntelligent Knee Stability Restoration (IKSR) robotic system and dedicated ACL reconstruction software (Droidsurg Medical Co., Ltd, Shanghai, China) (Figure 1A). The IKSR robotic system consists of a master control trolley, robotic arm, optical tracking system, footswitch, navigation and positioning system software, and accessory kits. It utilizes optical tracking and positioning technology to collect the positional data of the optical marker bodies on each component for intraoperative surgical planning. The robotic arm is controlled to perform movements to reach the location of the planned surgical access. After system calibration, 3D surface model of femur and tibia were generated by several anatomic landmarks, which were pointed by handheld touch probe with reflective markers. Two rigid bodies were fixed to the tibia and femur approximately 15 cm away from the tibial plateau and femoral condyle (Figure 1B). Each rigid body has a distinct conformation of reflective markers that can be tracked by the infrared tracker.

### 2.2 Planning of tunnel positions

**FIGURE 2:**
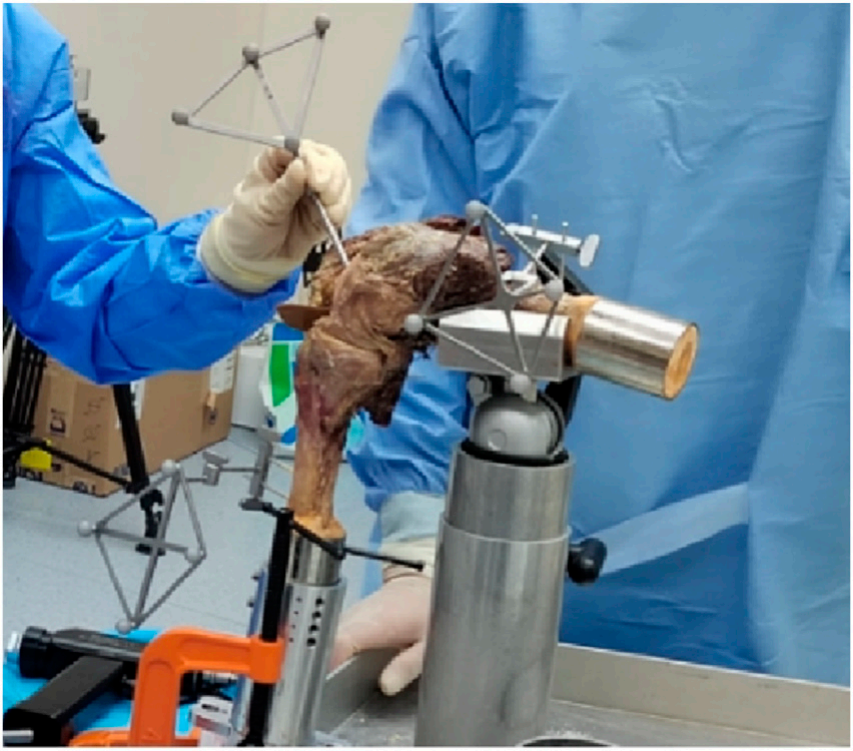
Marking the target tunnel position using a tracked touch probe with infrared tracer marker.

**FIGURE 3:**
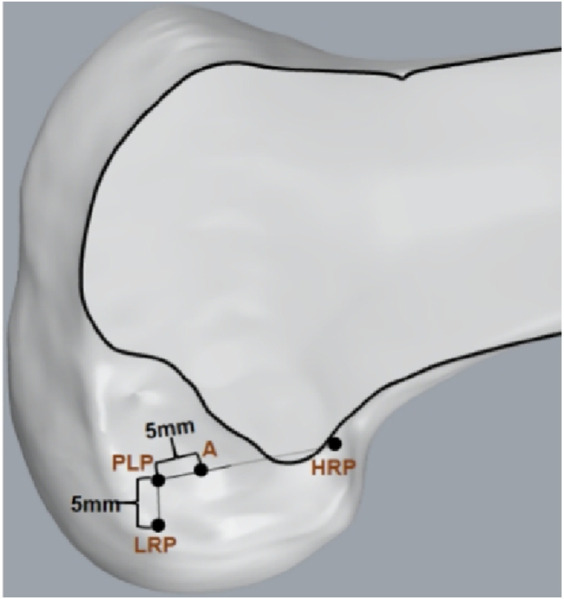
Femoral tunnel positioning (Point A) for the modified transtibial technique. (HRP, high reference point; LRP, low reference point; PLP, a point 5 mm anterior to the LRP; Point A, a point 5 mm to the PLP).

The knee was fixed to the operating table at 90° flexion. Ensure that the tibia was placed in a neutral position relative to the femur when the fixator was secured. No extra coronal or transverse plane torques were applied. The ACL attachment points were exposed in the joint cavity under direct observation, and the center of ACL footprint were identified. A tracked touch probe with infrared tracer markers was used to mark the target tunnel position and to perform accurate 3D reconstruction of points (Figure 2). For the modified TT technique, the tibial and femoral tunnel positions were determined according to the previous study describing an anatomical TT technique for ACL reconstruction (Zhao, 2020). The tibial tunnel was located at the intersection of the midline between the 2 transverse lines passing through the anterior edge of the anterior horn of the lateral meniscus and the lateral tibial eminence and the midline between 2 longitudinal lines passing through the base of its medial slope and the ridge of the medial tibial eminence. Two reference points, namely, the high reference point (HRP, i.e., the over-the-top point) and the low reference point (LRP, i.e., the lowest point of the lateral wall of the femoral notch) onto the surface of the femoral intercondylar notch, were determined to define the femoral tunnel position (Figure 3) (Zhao, 2020). PLP (posterolateral bundle point) was defined as a point 5 mm anterior to the LRP, and the femoral tunnel was located at a point between the PLP and HRP, with a distance of 5 mm to the PLP. For the AM portal technique, the anatomical area of the ACL insertion was considered as an accurate reference location for tunnel drilling. The femoral and tibial tunnel positions were located within the center of ACL footprints with the knee at 120°of flexion for this method (Trofa et al., 2020).

### 2.3 Navigated K-wire drilling

Once the centers of the tibial and femoral tunnel positions have been planned, the navigated drilling was initiated using the robotic arm. After the mark points were registered on the main console, the robotic arm with 7 degrees of freedom navigated based on the master station plan of the route and moved to the target position accurately. The cannula was inserted, and the tunnel center position was drilled using a 2.4 mm K-wire. The guide pin was passed from tibia into the femur, until it formed a line connecting the inner aperture of tibial tunnel to the inner aperture of femoral tunnel. For the AM portal technique, the robotic arm was placed on the lateral aspect of the femur. The 2.4 mm guide pin was inserted through the extra-articular point to the inra-articular point.

### 2.4 Data acquisition

The robotic arm tried to drill a tunnel according to the preoperative planning, however, the actual drilled tunnel (the intraoperative tunnel) could be different due to human error and systemic error. To assess the accuracy of the surgical navigation robot, we marked the preoperative and postoperative femoral tunnel position. The position of the preoperatively planned tunnel was identified and marked with colored markers. Postoperative obtained tunnel was used as the reference for evaluation of the accuracy of the system. The difference of preoperatively planned and postoperatively obtained femoral tunnel positions was measured directly on the cadaveric knee. Comparison of the preoperative planning and postoperative femoral tunnel positions showed that the mean difference of the tunnel position was 1.8 ± 0.4 mm.

**FIGURE 4:**
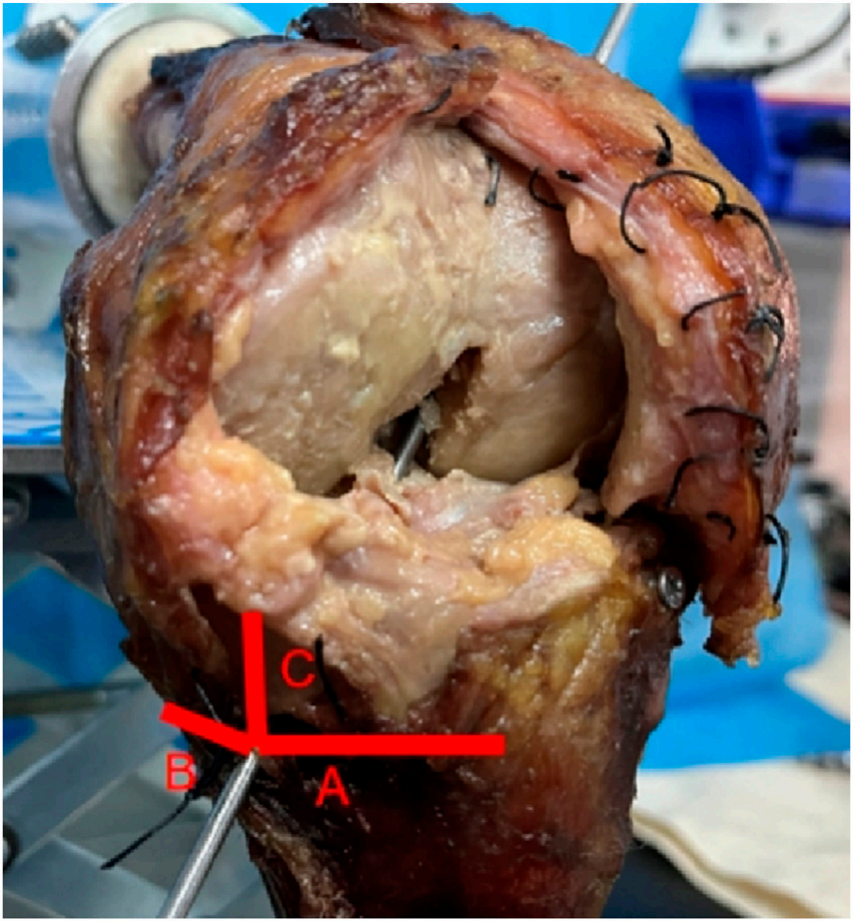
Tibial tunnel starting position. Line A distance from the outer aperture of tibial tunnel to the tibial anterior tuberosity; Line B distance from the outer aperture of tibial tunnel to the medial end of the tibia; Line C distance from the outer aperture of tibial tunnel to the tibial articular surface.

After navigating K-wire positioning into the planned tunnel positions, data acquisition of the tunnel aperture was performed. Measurements of the tibial tunnel position included the following parameters: distance from the outer aperture of tibial tunnel to the tibial anterior tuberosity, distance from the outer aperture of tibial tunnel to the medial end of the tibia, distance from the outer aperture of tibial tunnel to the tibial articular surface (Figure 4). Distance from the inner aperture of the tibial and femoral tunnel to the outer aperture of the tibial and femoral tunnel was defined as the tibial and femoral tunnel length.

**FIGURE 5:**
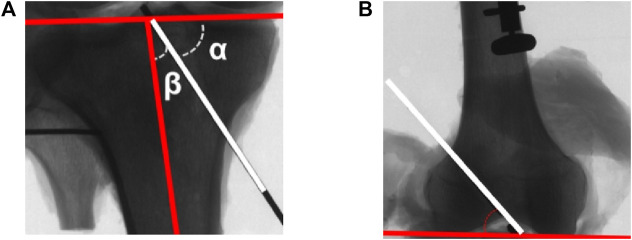
**(A)** The coronal angle (α) and axial angle (β) of the tibial tunnel (white line: tibial tunnel); **(B)** The coronal angle of the femoral tunnel (white line: femoral tunnel).

**FIGURE 6:**
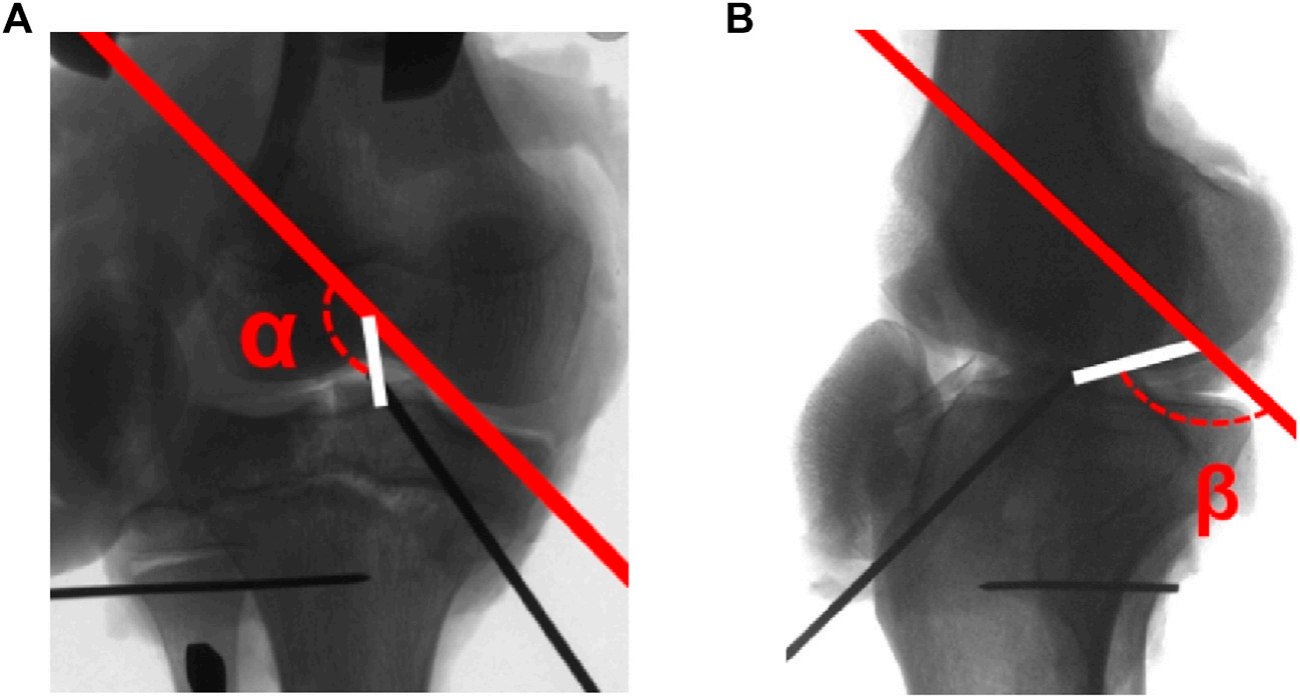
**(A)** The femoral tunnel-graft angle (α) in the coronal plane; **(B)** The femoral tunnel-graft angle (β) in the sagittal plane at full knee extension (red line: femoral tunnel; white line: graft connecting the inner aperture of tibial tunnel and femoral tunnel).

Fluoroscopic images of postoperative knee in anteroposterior and lateral projections were acquired with the K-wire determining the tunnel and graft obliquity. For the tibial tunnel, the coronal angle was defined as the angle between the tibial tunnel and the plane of the tibial plateau (Figure 5A), and the axial angle was defined as the angle between the tibial tunnel and the tibial long axis (Figure 5A). For the femoral tunnel, the coronal angle was defined as the angle between the femoral tunnel and the line tangent to the medial and lateral femoral condyles (Figure 5B). Graft obliquity was determined by measuring the femoral tunnel-graft angle of the coronal and sagittal plane at full knee extension (Figure 6).

### 2.5 Statistical analysis

All statistical analyses were performed using SPSS software (version 14.0, SPSS). The Shapiro-Wilk test was used to confirm the normal distribution of different variables. Paired sample *t*-test was used to compare the tunnel and graft characteristics between two tunnel drilling techniques. *p* values < .05 were considered statistically significant.

## 3 Results

### 3.1 Tibial tunnel starting position

For the modified TT technique, the distance from the outer aperture of tibial tunnel to the tibial anterior tuberosity was 17.3 mm ± 2.2 mm, the distance from the outer aperture of tibial tunnel to the medial end of the tibia was 24.0 mm ± 4.1 mm, and the distance from the outer aperture of tibial tunnel to the tibial articular surface 19.6 mm ± 5.4 mm (Table 1).

**TABLE 1:** Analysis of tibial tunnel position and tunnel length.

	Modified transtibial technique	Anteromedial portal technique	*p*-Value
Position of the outer aperture of tibial tunnel, mm			
To the tibial anterior tuberosity	17.3 ± 2.2	-	-
To the media end of the tibia	24.0 ± 4.1	-	-
To the tibial articular surface	19.6 ± 5.4	-	-
Tibial tunnel length, mm	34.0 ± 3.3	40.3 ± 2.3	<0.001
Femoral tunnel length, mm	42.0 ± 6.1	34.8 ± 4.5	<0.001

#### 3.1.1 Tunnel length

The mean tibial tunnel length of the modified TT technique was significantly shorter than in the AM portal technique (34.0 mm ± 3.3 mm vs. 40.3 mm ± 2.3 mm, *p* < 0.001). The mean length of the femoral tunnel was significantly longer for the modified TT technique than for the AM portal technique (42.0 mm ± 6.1 mm vs. 34.8 mm ± 4.5 mm, *p* < 0.001) (Table 1).

#### 3.1.2 Tunnel and graft obliquity

The mean coronal angle of the tibial tunnel was 48.9° ± 4.7° for the modified TT and 65.8° ± 6.5° for AM portal technique, with significant difference (*p* < 0.001). The mean axial angle of the tibial tunnel was significantly lower for the AM portal technique than for the modified TT technique (26.7° ± 3.1° vs. 44.9° ± 3.7°, *p* < 0.001). The mean coronal angle of the femoral tunnel was significantly lower for the AM portal technique than for the modified TT technique (46.5° ± 4.1° vs. 54.0° ± 4.7°, *p* < 0.001) (Table 2).

**TABLE 2:** Analysis of tunnel and graft obliquity.

	Modified transtibial technique	Anteromedial portal technique	*p*-Value
Tibial tunnel angle, °			
Coronal angle	48.9 ± 4.7	65.8 ± 6.5	<0.001
Axial angle	44.9 ± 3.7	26.7 ± 3.1	<0.001
Femoral tunnel angle, °	54.0 ± 4.7	46.5 ± 4.1	<0.001
Femoral tunnel-graft angle, °			
Coronal angle	139.5 ± 5.6	148.1 ± 7.4	<0.001
Sagittal angle	118.8 ± 5.2	128.9 ± 6.7	<0.001

A comparison of the femoral tunnel-graft angle at full extension in the two techniques found significant differences in both the coronal (*p* < 0.001) and the sagittal (*p* < 0.001) planes. The AM portal technique resulted in a graft bending angle that was significantly more angulated in the coronal (139.5° ± 5.6° vs. 148.1° ± 7.4°) and the sagittal planes (118.8° ± 5.2° vs. 128.9° ± 6.7°) compared with the modified TT technique (Table 2).

## 4 Discussion

This study directly compared anatomic SB ACL reconstructions performed with a modified TT technique and an AM portal technique. The results suggested that the perforating angle and location of guide wires could be adjusted to achieve anatomic TT reconstruction. The modified TT technique showed a larger femoral tunnel angle in the coronal plane and lower femoral tunnel-graft angle than the AM portal technique. The modified TT technique created a shorter tibial tunnel length but a longer femoral tunnel length than AM portal tunnels.

If anatomical femoral tunnel is desired using the TT technique, the corresponding tibial tunnel entrance must be identified. This study found an optimum starting position approximately 24.0 mm ± 4.1 mm medial to the tibial tubercle and 19.6 mm ± 5.4 mm distal to the tibial plateau edge. However, previous studies have presented different starting points of the tibial tunnel for anatomic TT reconstruction. Heming et al. (2007) used a cadaveric model to demonstrate the plausibility of the anatomical footprint using the TT technique, but commented that starting point of the tibial tunnel is unacceptably close to the joint line (less than 10 mm). Piasecki et al. (2011) found an optimum starting position 9 mm posteromedial to the tibial tubercle and 16 mm distal to the medial tibial plateau edge. Morgan et al. (1995) suggested starting the tibial tunnel 15 mm medial to the tibial tubercle and 10 mm superior to the pes anserinus. The present study explored more practical tibial starting point than the ideal trajectory of the previous studies. This is because anatomic femoral tunnel locations were modified in this study, which reduced tibial axial angulation and resulted in a more practical tibial starting point. In the present study, the femoral tunnel position was determined according to the previous study (Zhao, 2020). The high reference point and the low reference point onto the surface of the femoral intercondylar notch helped to define the femoral tunnel position (Zhao, 2020). Surgical navigation robot benefits surgeons by providing visual bony anatomy inside the surgical field and good results for tunnel orientation and position in accuracy and reproducibility (Lee et al., 2016). An ACL reconstruction robotic positioning system based on anatomical characteristics provides more accurate bone tunnel positioning compared to handheld locators (Ding et al., 2022). Future research would include the traditional hand-held locator group to provide valuable insights into the advantages of robotic-assisted ACL reconstruction techniques. The implication of this study is that a more distal and medial tibial tunnel entrance will result in anatomic graft positioning with the assistance of surgical navigation robot.

In this study, the recommendation to place the tibial guide pin at 49° to the tibial plateau and at 45° to the tibial long axis represented an anatomic tunnel position. Our results show that the tibial plateau angle and tibial long axis angle vary within a certain range among different subjects, with standard deviations of 4.7° and 3.7° respectively. These individual differences have a minor influence on our experimental conclusions. In clinical practice, surgeons need to make small adjustments based on each patient’s specific anatomy to achieve optimal surgical outcomes. This is in agreement with the work of Heming et al. who indicated that a 47.9° tibial tunnel angle to the tibial plateau is necessary to achieve anatomic femoral tunnel in the TT ACL reconstruction (Jennings et al., 2017). In a previous cadaveric study, the guide pin was placed at the center of tibial and femoral footprints, showing that the tibial tunnel angle was 42.1°to the tibial shaft (Heming et al., 2007). Inconsistent with our results, (Howell et al., 2001) recommended an anatomic position of the femoral tunnel if the tibial guide pin was placed at 60°–65° to the tibial plateau. The findings of our study and previous studies defend the criteria defined by Howell, and favor the idea that the coronal tibial angle should be much lower, in order to place the femoral tunnel in the right place. For anatomical ACL reconstruction under arthroscopy, the TT technique has difficulty in determining appropriate tibial tunnel angle and achieving accurate tunnel position (Segawa et al., 2005). Our data indicates tighter control over tibial tunnel placement may translate to more consistent graft placement and better restoration of anatomic characteristics after reconstruction. Clinical adoption of these findings could reduce graft failure rates and the need for revision surgeries. In this study, it was sought to identify the clinical availability of the surgical navigation robot with emphasis on attainment of appropriate tunnel angle, as well as accurate localization for tunnelling. Previously, anatomical reconstruction may not be achieved using the modified TT technique during arthroscopic procedure due to human factors. The surgical navigation robot could minimize the human error, and the result of the present study showed that the mean difference of the preoperative planning and postoperative femoral tunnel positions was 1.8 ± 0.4 mm. It appears that surgical navigation robot combined with the modified TT technique can achieve the desired tunnel position for anatomic ACL reconstruction.

Our study found that the coronal femoral tunnel angle created by the AM portal technique were more horizontal than those of the modified TT technique (46° vs. 54°). In agreement with our finding, (Youm, 2014) reported the difference in coronal obliquity of the femoral tunnel was about 7° between the AM portal technique (42.5°) and the modified TT technique (49.3°). Bedi et al. (2010) reported that the coronal angles of the oblique femoral tunnels from either the AM portal or the TT techniques were 45.9° and 54.1°, respectively. Additionally, our coronal angle of the femoral tunnel in the modified TT technique was on average 54.0° ± 4.1°, and it was lower than the results (61.7° ± 5.5°, 58.8° ± 8.3°) of traditional TT techniques reported by previous studies. This indicated that anatomic TT ACL reconstruction compared with the traditional TT technique created a more oblique femoral tunnel. The modified TT technique maintained a comparably favorable degree of angulation, which would be expected to make graft passage of similar ease as with the traditional TT technique. Previous studies have concluded that the horizontal trajectory of the guide wire may increase the risk of posterior cortical breakthrough (Bedi et al., 2010). Thus, not only is the modified TT technique more reproducible, but it is also safer when such complications of the AM portal technique is taken into consideration.

The AM portal technique resulted in femoral tunnel-graft angle that was much more angulated than the modified TT technique in the coronal (139.5° vs. 148.1°) and the sagittal planes (118.8° vs. 128.9°). A cadaveric model demonstrated that the increased femoral tunnel-graft angle was associated with the increased graft strain (Sinha et al., 2015). It has been reported that with the greater tunnel-graft angle, the greater force on the graft at the tunnel aperture as it is stretched over this bony edge (Ahn et al., 2016). Relative to the traditional TT tunnels, the increased graft obliquity in the AM portal technique would be predicted to increase force by 98.3% (29° increase in tunnel-graft angle) versus 27.6% (8° increase in tunnel-graft angle) for the modified TT technique. Additionally, acute graft bending angle may be a critical biomechanical factor contributing to poor graft immaturity or graft failure (Ahn et al., 2017; Tashiro et al., 2017). This suggested that the AM portal technique compared with the modified TT technique resulted in more acute bending of the ACL graft at the femoral tunnel aperture, which may be associated with postoperative complications such as femoral tunnel expansion and graft immaturity or damage.

The mean tibial tunnel length of the modified TT technique was significantly shorter than in the AM portal technique (34.0 mm vs. 40.3 mm). The mean length of the femoral tunnel was significantly longer for the modified TT technique than for the AM portal technique (42.0 mm vs. 34.8 mm). Tibial tunnel length shorter than 30 mm and femoral tunnel length shorter than 35 mm may compromise graft fixation and tunnel-graft length match (Loh et al., 2003). A previous cadaveric study reported that a tibial tunnel created with proximal starting point would result in tunnel-graft mismatch problems and compromise tibial graft fixation. A study found that a shorter femoral tunnel length at certain angles was significantly associated with a shorter distance between the tunnel and the medial femoral condyle (Loh et al., 2003). Another study investigated the position of the femoral tunnel after tunnel widening and shifting (Lee et al., 2016). It found that the tunnel center and margins shifted anteriorly, indicating potential changes in tunnel length (Lee et al., 2016). Ebersole et al. (2016) commented that the femoral tunnel length was considerably shorter than 35 mm for an accurately positioned footprint, which may result in unstable fixation. However, this study has proved that a relatively distal and medial starting position resulting in anatomic tunnels is practical and achieve a longer tibial tunnel and femoral tunnel length. One issue to be aware of during ACL reconstruction is the length of the autograft tendon. To achieve the fastest and best tendon-bone healing, there is an optimal length, with placement in the bone tunnel equal to 17 mm or more. Therefore, the length of the autograft tendon is suitable for the tunnel length created by the modified TT technique.

The limitation of this study was that the number of cadaveric knees was small. Graft strain was not measured in this study, therefore, it is not yet clear of the relative role that aperture position and tunnel angulation play on graft strain after anatomic ACL reconstruction. The gender-based relationship between joint function and outcome after ACL reconstruction conducted by surgical navigation robot remains unclear and requires further clinical investigation. The present study did not measure graft strain, and future study should include the measurement of graft strain. While cadaveric studies offer valuable insights, further clinical trials involving living patients are necessary to validate the findings in a real-world setting. The modified TT ACL reconstruction was performed in cadaveric knees, and long follow-up will be needed in future clinical practice to investigate the clinical and biomechanical outcomes.

## 5 Conclusion

The surgical navigation robot has the potential to accurately identify and drill bone tunnels in anatomic ACL reconstruction. This study demonstrated the practical perforating angle and location of guide wires for anatomic reconstruction using the modified TT technique conducted by surgical navigation robots. These findings indicate that both the modified TT and AM portal techniques could achieve anatomic tunnel placement but that the tunnel direction differed significantly. Compared with the modified TT technique, the more horizontal trajectory of the femoral tunnel in the AM portal technique creates a shorter femoral tunnel length and a more acute graft bending angle, which may negatively affect graft fixation and healing. Therefore, it is possible to modify the TT technique to achieve more anatomic ACL reconstruction, and the modified TT technique has benefits in a relatively long femoral tunnel length, smaller femoral tunnel and graft obliquity, which leads to stable graft healing.

## Data Availability

The original contributions presented in the study are included in the article/Supplementary Material, further inquiries can be directed to the corresponding authors.
